# Unexpected Complications of Novel Deep Brain Stimulation Treatments: Ethical Issues and Clinical Recommendations

**DOI:** 10.1111/ner.12613

**Published:** 2017-05-30

**Authors:** Hannah Maslen, Binith Cheeran, Jonathan Pugh, Laurie Pycroft, Sandra Boccard, Simon Prangnell, Alexander L. Green, James FitzGerald, Julian Savulescu, Tipu Aziz

**Affiliations:** ^1^ The Oxford Uehiro Centre for Practical Ethics University of Oxford Oxford UK; ^2^ Oxford Functional Neurosurgery University of Oxford Oxford UK; ^3^ Psychological Medicine Oxford University Hospitals, Oxford UK

**Keywords:** Chronic pain, complications, consent, deep brain stimulation, seizures

## Abstract

**Background:**

Innovative neurosurgical treatments present a number of known risks, the natures and probabilities of which can be adequately communicated to patients via the standard procedures governing obtaining informed consent. However, due to their novelty, these treatments also come with unknown risks, which require an augmented approach to obtaining informed consent.

**Objective:**

This paper aims to discuss and provide concrete procedural guidance on the ethical issues raised by serious unexpected complications of novel deep brain stimulation treatments.

**Approach:**

We illustrate our analysis using a case study of the unexpected development of recurrent stereotyped events in patients following the use of deep brain stimulation (DBS) to treat severe chronic pain. Examining these unexpected complications in light of medical ethical principles, we argue that serious complications of novel DBS treatments do not necessarily make it unethical to offer the intervention to eligible patients. However, the difficulty the clinician faces in determining whether the intervention is in the patient's best interests generates reasons to take extra steps to promote the autonomous decision making of these patients.

**Conclusion and recommendations:**

We conclude with clinical recommendations, including details of an *augmented consent process* for novel DBS treatment.

## Introduction

Deep brain stimulation (DBS) treatments, particularly when they are novel, raise new challenges for clinical ethics and clinical protocol. Patients often will initially consent to novel DBS treatments with limited information about potential complications associated with that particular intervention (for that target site, for that indication, for that sort of patient), due to its investigational nature. The limited information patients do have does not generalize consistently, given individual differences in brain structure and function. How the consent process for novel DBS treatments should present information to prospective DBS patients about any serious unexpected complications emerging in the existing individual cases needs attention. Novel DBS treatments are not entirely exceptional among neurological interventions in the challenges they raise. However, the increasing use of DBS in novel targets, with the potential for irreversible consequences, compounds the need to ensure there are adequate procedures to support patient decision making. This is most acute in the context of complications with unknown incidences and uncertain implications. Novel DBS treatments thus constitute a priority category of interventions for revisiting the ethical requirements for patient consent.

DBS is a nonablative neurosurgical procedure that has been used to ameliorate symptoms of neurological conditions, including Parkinson's Disease 1, dystonia 2, and chronic pain 3. Although DBS is a powerful therapeutic tool, it also involves a degree of risk. DBS involves a neurosurgical intervention with perioperative risks of haemorrhage (1.3–4%), epileptic seizures (0.4–2.8%), and pneumonia (0.4–0.6%), among others 4, 5. The long‐term presence of brain implants also brings risks of infection (2.8–6.1%), lead migration or misplacement (5.1%) and skin erosion (1.3–2%) among others 4. Finally, stimulation itself has been associated with an array of adverse cognitive, behavioral, psychiatric and psycho‐social side‐effects, depending on the targeted brain area 4, 6.

As is the case with any medical intervention, it is crucial that patients adequately understand the nature and degree of the risks attending the intervention, in addition to the likelihood of therapeutic benefit, prior to consenting to treatment. This is to some extent less problematic in the context of carrying out DBS on well‐established targets for clear indications, since the patient's decision can be informed by existing data about the attendant risks and the impact that adverse outcomes can have on patients' lives (although unexpected complications can sometimes arise even in the context of well‐established DBS treatments) 7, 8. However, when unexpected complications occur in DBS therapies employing novel targets for new indications, there is no existing data to indicate whether any complications are a unique occurrence, or if they are likely to be experienced by a large number of patients undergoing stimulation. How this feature should be addressed in the consent process is a pressing question. Further, this uncertainty makes clinical determination of what is in a particular patient's best interests very difficult.

In this paper, we discuss the ethical issues raised by serious unexpected complications of DBS by focusing on a case study of the unexpected development of recurrent stereotyped events in a number of patients following the use of DBS in the treatment of severe chronic pain. We conclude with clinical recommendations, including a new protocol for an *augmented consent process* for novel DBS treatment.

## Seizures After Anterior Cingulate Cortex DBS

Medically refractory severe chronic pain can be debilitating, with a severe impact on a person's quality of life. Although DBS of targets like the sensory thalamic nuclei 9 and periventricular gray 3 are relatively well established for well‐localized pain (e.g., segmental poststroke pain), an effective target for poorly localized widespread medically refractory pain has, until recently, proven more evasive.

The anterior cingulate cortex is an established target for pain relief in terminally ill patients, most commonly pain associated with terminal cancer 10. The established procedure, called cingulotomy, is destructive lesioning of the anterior cingulate cortex (ACC). It is successful in around 50% of patients, but side‐effects are common, and occur even in those who do not benefit from pain relief. DBS has several advantages over lesioning. The side‐effects of DBS are considered reversible, operative complication rate is low, and DBS can be “dosed” as symptoms change. DBS of the ACC was, therefore, an option for those with nonterminal illness, unlike lesioning of the ACC. The ACC DBS implant program for medically refractory pain was pioneered in Oxford by the Functional Pain Neurosurgical team under TA. As a (novel) public health‐funded (on grounds of exceptional need) treatment, performed as clinical intervention, the program did not require research ethics review (correspondence to TA from REC, via UoX Senior Clinical Research Support Manager).

The first patient was implanted on February 21, 2007. There was a low rate of success during the week of trial stimulation initially. With the recognition that bilateral implants, and higher stimulation parameters were required, and with refinement of surgical targeting, results from late 2011 onwards indicated that 14 of 16 patients (87.5% response rate) reported appreciable benefit during the week of trial stimulation, and went on to full implantation.

Pain is a complex symptom with at least three dimensions contributing to its experience 11: the sensory dimension consists in one's awareness that one is in pain, how intense it is, where it is located, and so on; the affective dimension consists in the experienced unpleasantness, as well as emotional responses such as fear or anger; the cognitive dimension consists in thoughts about the pain, such as “I should go to the doctor” or “this is ruining my day.”

Stimulating the ACC seems to principally affect the affective dimension of pain: patients were still aware that they were in pain, but no longer found it aversive. Indeed, several patients described feeling as if their pain was separate from them physically and said that they did not think about it anymore, although they could still sense it 12.

The outcomes of the ACC DBS program were published by Boccard et al. 12. In keeping with the hypothesis that ACC DBS targets the affective dimension of pain, improvements on the visual analogue scale for pain did not correlate with the more significant improvements in quality of life, as measured by the EQ‐5D. The significant improvements in quality of life are striking in a patient cohort with medically and surgically refractory pain (including in several cases DBS of established pain targets like sensory thalamic nuclei).

The success of this procedure was tempered by the onset of recurrent stereotyped events in several patients. Some patients experienced recurrent seizures, while two developed de novo epilepsy. The Surgical Implant team or referring clinicians at other centers treated these events over a two‐year period. The implant team halted the ACC DBS program and requested a review of the ACC program by the DBS Neurology service in 2014. Following review of medical notes and telephone interviews using a validated questionnaire, six patients were identified to have suffered recurrent stereotyped events suggestive of seizures. In one, events were felt to related to alcohol abuse/withdrawal, and the patient declined specialist review. In another, events had ceased following explantation (following infection), and the patient has declined follow up. Four patients were identified to be suffering ongoing events, and assessed by DBS Neurology service with clinical evaluation and EEG video telemetry in 2015. A full clinical paper detailing the outcomes of this evaluation is in preparation. Below, we summarise key outcomes.

Patient one developed recurrent stereotyped neurological events (“difficulty finding words”) above a certain (previously well tolerated) threshold of stimulation intensity, 12 months after surgery. By the time, he first reported symptoms he had reduced stimulation intensity. He recognized that the symptoms occurred above a certain threshold, and that the threshold was falling over time. Ramping up DBS stimulation intensity to above a certain threshold could reliably reproduce symptoms and electrographic changes. Patient switched off the stimulator as pain relief was felt to be minimal at safe stimulation intensities. Seizures stopped occurring once the stimulation was switched off, but the pain increased to preoperative levels within weeks. Patient two suffered convulsive seizures at the very high stimulation intensities required for near‐complete pain relief, in the immediate postoperative period. Stimulation intensities just below the convulsive threshold were well tolerated for nearly two years before Patient two reported stereotyped neurological events (“lip smacking”). Patient two refused testing of stimulation thresholds above those he recognized as safe during video‐telemetry, but like Patient one, has now switched off DBS as the threshold for seizures has fallen below that required for useful pain relief.

Patients three and four had a similar onset to the first two patients, but stimulation intensity was not turned down. Patient three could not tolerate pain, and increased stimulation on his own or resisted turning down stimulation. Patient four's seizures were unrecognized for some time, and even after seizures were recognized. Patient four could not tolerate stimulation being turned down. Patients three and four showed progressive increases in seizure frequency and severity, with patient four eventually suffering generalized convulsive seizures, possibly amounting to status epilepticus. This occurred despite being on antiepileptic medication, and despite stimulation being turned off. Thus, while Patients one and two have stimulation‐induced seizures, Patients three and four developed *de novo* stimulation induced epilepsy (Table 1).

**Table 1:** Evaluation of Treatment and Outcomes for Four of Six Patients Suffering Seizures.

	Indication for DBS	Onset of seizures	Semiology	Inter ictal EEG	Ictal EEG	Stimulation parameters at onset of seizures	Fate of DBS
Patient 1	Poststroke pain (Haemorrhagic stroke on the right)	12 months	Affective seizure (Psychic aura) and Speech arrest Brought on by rapid ramping of stimulation	Intermittent, infrequent sharp transients over the frontocentral regions.	Symmetrical frontal delta wave activity in long runs	3.5 mA 130 Hz 450 mics	Discontinued but felt to be worthwhile even at low settings
Patient 2	Poststroke pain (Right side stroke)	24 months 2 GTC seizure with suprathrehold stimulation in immediate postoperative period.	Loss of awareness with lip smacking (Automotor seizures) Two generalized tonic clonic seizures reported	Fast activity is prominent over the vertex	No electrographic change	8 mA 130 Hz 450 mics	Discontinued
Patient 3	Postsurgical pain on the right following Spinal surgery for syringomyeila	12 months	Left arm tonic seizures with speech arrest Minor jerks in the left leg sometimes (Left leg myoclonic seizures)	Sharp transients are noted intermittently, maximal over both frontal regions, and occasionally focal in the right superior frontal area	No electrographic change	6 mA 130 Hz 450 mics	Discontinued, then resumed (patient request) Following management of seizures. Discontinued again following seizure recurrence
Patient 4	Whole spine pain‐failed back surgery	20 months	Dialeptic (Behavioural arrest with loss of awareness) seizures with secondary generalization (generalized seizures are nocturnal)	Sharp wave discharges are seen across the fronto‐central mid‐line with right sided emphasis	Rhythmical, symmetrical 6 Hz theta slow wave activity across the fronto‐central regions that increments in amplitude and slows in frequency before ending abruptly	5V 130 Hz 450 mics	Discontinued, then resumed (patient request) Following management of seizures. PC&S device (see later in text) implanted.

## Principles in Medical Ethics

The four principles of medical ethics are well established 13. A clinician must consider the ethical permissibility of certain acts, guided by the principles of nonmaleficence, beneficence, respect for patient autonomy, and justice. Nonmaleficence requires that the clinician does no harm to the patient; beneficence requires the clinician to act in the patient's best interests; autonomy requires that the clinician act in line with the expressed autonomous preferences of the patient; justice requires clinicians to act in a way that is fair, in the context of the wider population of patients. These principles sometimes come into conflict. Working out what to do requires careful balancing of the ethically relevant considerations. In the interests of brevity, we shall focus only on the first three principles, bracketing considerations of justice in this context. Justice determines what resources are available for the use of medical procedures in defined patient groups. This typically uses cost‐effectiveness analysis and some theory of distributive justice, such as utilitarianism or egalitarianism. We focus here on what is ethically required for individual patients, as such requirements must be met in any distributive system, and can be discussed independently of broader considerations of the global patient population.

### Nonmaleficence

Nonmaleficence requires that the clinician does no harm to the patient, and is often seen as the primary duty of a doctor: “First, do no harm.” However, it is best considered together with beneficence in determining what is in a patient's best interests: whether the expected benefits outweigh the expected harms.

Since ACC DBS‐induced seizures could plausibly be seen as directly harmful to the patient, and because ACC DBS might cause a distinct, further medical condition, the principle of nonmaleficence might be thought to weigh decisively against offering the treatment; this is especially so when the new condition is associated with further risks (in extreme cases, perhaps of accidents or sudden unexplained death from epilepsy that may be greater than those accompanying chronic pain).

However, risk of seizures is not the only relevant consideration. The purpose of medicine is not restricted merely to physiological healing and survival. Indeed, the relief of pain is one of the primary goals of medicine. Intractable chronic pain significantly reduces the patient's quality of life, and ACC DBS was shown to improve quality of life for some patients, notwithstanding some adverse events. Further, depending on the patient, chronic pain might pose a significant indirect risk to life if it leads to depression and suicidal ideation. In such cases, the threat to life posed by the DBS surgery and any resulting seizures will not necessarily be greater than the indirect threat to life presented by chronic pain. The principle of nonmaleficence, then, does not weigh decisively against offering ACC DBS.

### Beneficence

Beneficence requires the clinician to act in the patient's best interests. The determination of best interests can, however, be complex. The evaluation should not only consider medical interests, but also the patient's overall interests or well‐being, which have objective and subjective elements. That is, some things will be good or bad for all patients, while others will be good or bad depending on (and as a consequence of) the patient's desires and values. Where justice allows the offering of a procedure, and that procedure is not clearly against the patient's interests, the patient should be offered the procedure and assisted in her assessment of whether the benefits outweigh the risks, according to her goals and life plans. Accordingly, beneficence and respect for autonomy proceed in concert.

Given the magnitude of the risks associated with ACC DBS, it is not straightforward to determine whether it is in patients' best interests, generally speaking. As noted above, the likely effect on the particular patient's quality of life will be a central consideration, and the patient will often be best placed to assess how particular changes would affect her quality of life. Indeed, it is notable that Patient three continued using DBS as the pain was so severe, despite having developed epilepsy. Patient one discontinued but observed that DBS was effective. Differences in the overall effect of the intervention on these patients' quality of life may have factored into their divergent decisions regarding continuation of treatment.

However, even knowing the patient's view on the likely effects on her wellbeing, it may still not be possible for the clinician to determine whether the intervention would be in her best interests: the significance of the risks, and whether they are worth taking is something the patient must decide for herself. Given this, the need to prioritize patient autonomy in this context becomes clear: while it is not the case that undergoing ACC DBS for chronic pain is clearly *contrary* the patient's best interests, the uncertainty is such that the intervention can be offered for the patient to consider, but not recommended.

### Respect for Autonomy

Given the uncertainty about whether ACC DBS will overall benefit the patient, there is a need to ensure that patients who opt to undergo the procedure choose to do so autonomously. The significant clinical challenge, then, will be to make sure that the consent process and related decision‐making circumstances facilitate autonomous choice.

Beauchamp and Childress provide the standard account of what is required for autonomous decision making (or action) 13. According to this account, aautonomous action is characterised “in terms of normal choosers who act 1) intentionally, 2) with understanding, and 3) without controlling influences that determine their action.”

This account is often supplemented with the additional criterion that autonomous choices must not be *irrational* (see e.g., 14, 15); on such views, individuals are most robustly self‐governing when they do not make errors in their reasoning. To make an irrational decision is to make a decision on the basis of error. We will focus on the errors that patients might make during the decision‐making process. Individuals can make two types of errors in their reasoning. First, they may make errors in their reasoning about *what to believe*. For instance, an individual may fail to give due consideration to available evidence that counts against a belief, and its logical or probabilistic implications. One of us (JS) has defended the claim that patient reasoning should also involve vivid imagination of what the consequences of their choices would be like *for them* 14. Second, individuals may make errors in their reasoning about *what to do*. For instance, an individual makes such an error if they choose to do something that impairs pursuit of a goal that they want to achieve. For example, if you think you need medical treatment and want to avail yourself of it, yet repeatedly choose not to go to the doctor, you act irrationally; you will fail to proceed toward your endorsed goal of acquiring medical treatment.

## Informed Consent and Patient Decision Making: The Case of ACC DBS

The decision‐making process for novel DBS treatments, including for chronic pain, must aim to avoid patients making errors in their decisions. As explained above, this is a prerequisite for patients' decisions to be autonomous. Where the intervention is not clearly in the patient's best interests, as is the case with ACC DBS for chronic pain, robust self‐governance is all the more important. Accordingly, we must consider whether patients might be susceptible to making either of the two types of errors of reasoning that we delineated above.

### Is the Patient Failing to Achieve Her Goal in Choosing DBS?

The principal question in relation to the assessment of the chronic pain patient's means‐end reasoning is whether treatment can be expected to be effective in relation to the patient's goal. The patient's goal might be construed narrowly (i.e., “to be pain free”) or more broadly (“i.e., to have a better quality of life”). More broadly construed, the net effect of the intervention on quality of life, including any reductions in this quality resulting from seizures must be considered in addition to the specific contribution made by the presence or absence of chronic pain.

In relation to the former construal, it can be expected that ACC DBS is likely to be effective in achieving the patient's goal. The data from the Oxford Implant Program demonstrated that most patients experienced a reduction in the aversive component of their pain, so were in an important sense free from pain, even if the awareness of pain was still present. Thus, if the patient's goal is construed in this way, it can be rational for the patient to choose to undergo ACC DBS, even given the risk of adverse side effects (assuming seizures are not expected to result in a significant degree of pain).

However, things are more complicated if we construe the patient's goal more broadly, as the aim to “have a better quality of life.” In relation to this goal, the means of ACC DBS may be more or less effective depending on the effects that seizures would have on the particular patient's wellbeing, and, relatedly, depending on the patient's values.

Although we would expect the patient's quality of life to be improved by ceasing to experience her pain as aversive, her quality of life may concurrently be decreased by the experience of seizures, and the broader implications for her life activities. The ways in which seizures compared with chronic pain affect a patient's quality of life, and which of these adverse experiences is the least bad will depend in large part on the individual patient, her goals and values. In the Oxford Implant Program, some patients chose to stop treatment because of seizures (despite effective pain relief), while others wanted to persist despite experiencing several seizures per day.

For example, imagine two patients who both have intractable chronic pain, and who are medically similar in all other relevant respects. One of these patients is a writer, while the other enjoys driving and the freedom to explore that driving gives her. The writer finds that her chronic pain prevents her from achieving the immersed state of mind that she needs in order to write. She fully appreciates the risks that attend DBS surgery, and has thought hard about how seizures, if present, would affect her life. Although she fears experiencing seizures, she feels confident that her quality of life would on balance be improved, since she would be able to focus again on her writing.

The driver, in contrast, is much less confident that the intervention would have a net positive effect on her quality of life. She realizes that experiencing seizures when driving would not only be very dangerous, but might result in her being legally prohibited from driving. She imagines her life without the aversive component of chronic pain but with the significant implications for her prospect of being able to continue driving when and wherever she wants, as she loves to do. She decides, on balance, that the net effect on her quality of life would not be positive enough to justify the intervention.

Although these examples are highly schematized, they serve to illustrate that whether ACC DBS is a sufficient means to achieve the goal of improved quality of life will differ from patient to patient, depending on what they value. This assessment will be further influenced by estimations of the likelihood of developing recurrent seizures, and the further likelihood that these seizures would cause significant harm. Taken together, these objective and subjective features feed into an assessment of the overall expected outcome of undergoing ACC DBS for the particular patient, and whether this expected outcome is positive enough to render the decision to undergo the intervention rational, or, indeed, in the person's best interests.

Patients' values are always relevant when a medical practitioner is working out what to offer and recommend. ACC DBS is not unique in this regard. However, the significant differences between the nature of seizures compared with chronic pain, and the varied impact each can have on individuals' lives make interrogation of patient values acutely important in this case. The lack of objective method to determine which is worse and what risks are worth taking does not mean that there is no way to determine what should be done. On the contrary, patients with all the materially relevant true beliefs will be able to give insight into what the implications would be for their lives.

However, to be in the position to give such insight, patients must not have made errors with respect to their reasoning about what to believe. If they are to weigh up whether or not the intervention is likely to improve their quality of life, they must have sufficient appreciation of the nature, implications, and likelihood of the intervention's consequences. We now turn to discussion of potential sources of error in relation to beliefs.

### Is the Patient's Reasoning About the Material Facts Subject to Error?

As noted above, one of us (JS) has previously argued that rational decision making requires vividly imagining what the consequences of our choices will be like *for us* 14. Fulfilling this condition in this context requires more than the patient's mere internalization of facts about the probability of seizures and so on; in addition, the patient must be able to vividly conceive of what *her* life would be like with seizures and associated risks.

John Stuart Mill famously defended autonomy, or individuality as he called it. Mill argued that each person has “privileged access” into her own life 16, so individuals should be the ones to decide what happens to them. Such privileged access may well be true of the evaluation of chronic pain—the patient herself may be best able to judge how bad the experience of the pain is. Indeed, third parties may underestimate the badness of another's chronic pain, and this may lead them to formulate misinformed risk‐benefit analysis of the treatment.

However, while patients often have privileged access to their own experiences, they are not incorrigible in evaluating their own interests. In particular, chronic pain patients may not be the best judges of the badness of seizures that they are at risk of experiencing following DBS, but which they have not yet experienced. If this is the case, patients will be susceptible to forming false beliefs in relation to the nature and significance of seizures and, correspondingly, the overall assessment of the risk of seizures as all‐things‐considered a risk worth taking. Such false beliefs could, in turn, steer a decision to opt for treatment that does not in fact serve their practical goal, as described above. Thus, the difficulty that the clinician has in determining the patient's best interests can sometimes extend to the patient herself. Where the patient's difficulty in this regard is concomitant with a failure to reason with materially relevant true beliefs, the patient's autonomous decision making is jeopardized.

Consequently, we must consider the possibility that chronic pain inhibits the vivid imagination of potential seizures and the development of epilepsy. For example, it is possible that the discomfort of chronic pain results in an asymmetric focus on what life would be like without the pain rather than on what life would be like with seizures. Crucially, this exacerbates the difficulty that any individual has in vividly imaging an entirely novel experience.

## Augmented Consent and Doctor–Patient Decision Making

Clinicians, therefore, face a challenge in working out how to maximize a patient's ability to imagine what living with seizures would be like, and to helping the patient determine whether the intervention is overall likely to improve her quality of life. Such information, when grasped, increases the quality of patients' consent. However, ensuring that patients have all the materially relevant information, and make decisions on its basis, is made harder by the fact that, at least at present, treatment is investigational and probabilities cannot be confidently assigned. As a corollary, it is also important that the risks are not overstated.

We, therefore, recommend an *augmented consent process*, to maximize the information the patient receives, and minimize the likelihood that her autonomy is undermined through failures in reasoning. We argue that such safeguards, as we set out in detail below, are most effectively implemented through adopting a liberal rationalist approach to doctor–patient decision making, which we outline now.

### Liberal Rationalism and the Role of Doctors and Surrogates in Patients' Decisions

One of us (JS) has previously defended a model of the doctor–patient relationship according to which doctors and patients should engage in a rational discussion about which course of action is best for the patient, all things considered 17. On this model of “liberal rationalism,” it is assumed that doctors and patients may not share the same set of values, and that neither party is incorrigible in their evaluative judgments. In view of these assumptions, although liberal rationalism advocates that patients should evaluate the available material information, and come to their own judgment of what is best, the model acknowledges that this process is best facilitated by the patient engaging with an empathetic and reasonable physician who is prepared to form their own understanding of what is in the patient's best interests.

In the light of epistemic uncertainty, discussion between the two parties about the rationales underlying their judgments allows for the best approximation of what is in the patient's best interests. To illustrate, in the present context, the physician may believe that DBS is in the patient's best interests because of the severe nature of the patient's pain. Conversely, the patient might believe that the risk of developing *de novo* epilepsy means that DBS is not in her best interests. In order to resolve this conflict, liberal rationalism would advocate that the physician and patient engage in a rational discussion about the justification for their views about what is in the patient's best interests. For instance, the physician may be able to further explain the nature of the risk that the patient faces, and what sort of effect epilepsy might have on the patient's life in comparison to her chronic pain.

In contrast, the patient may be able to provide further reasons for her judgment that the risk of developing epilepsy is not one that she is willing to take; perhaps this would threaten her capacity to continue in a particular profession in a way that her chronic pain does not. The inclusion of proxy decision making adds a further voice that may serve to highlight a further set of reasons to consider in the treatment decision. Perhaps the patient's spouse might provide a more accurate assessment of how well the patient is able to cope with her chronic pain in her daily life.

### The Augmented Consent Process for Novel DBS Treatments

In line with standard practice, all known possible side effects should be discussed with the patient when obtaining informed consent for any surgery, and investigational DBS neurosurgery is no exception.

In addition to identifying risks associated with all major operations and anesthesia, existing forms for patient information and consent for DBS already include sections on specific and general risks of DBS (Box 1).

**Box 1.** Cambridge University Hospitals (Oct 2013), “Patient information and consent to Insertion of a deep brain stimulator (DBS)”

**Specific risks of DBS**

**Short term adverse effects of stimulation**

These are common and usually reversible with adjustments in stimulation

- Eyelids closing
- Difficulty talking
- Limb or face contractions
- Jerky movements
- Visual flashes
- Numbness or tingling in limb or face
- Anxiety and nausea.

**Long term adverse effects of stimulation**

These problems have been reported in a small number of patients:

- Side effects caused by the stimulation as described above that the patient finds intolerable so that the stimulator is turned off
- Long term weight gain
- Depression
- In a small number of patients the equipment itself may break or fail.

**General risks of DBS**

The risks of surgery in general includes problems with the wound (e.g., infection) problems with breathing, such as chest infection and blood clots, for example in the legs and, less frequently, the lungs. According to published scientific literature these risks associated specifically with deep brain stimulation are:

- Blood clot in the brain causing permanent disability: two patients out of 100
- Stroke or other neurological deficit: one patient out of 100
- Infection of the leads: five patients out of 100
- Meningitis: less than one patient in 100
- Erosion of the wires through the skin: two patients out of 100
- Seizures: less than one patient in 100
- Weakening of the voice: five patients out of 100
- Short term confusion: five patients out of 100
- Death due to a blood clot: less than one patient in 100

However, where DBS surgery is investigational, and unexpected side effects (such as seizures) emerge in patients who have undergone the procedure, these particular side effects should be discussed in vivid detail with patients who are deciding whether to undergo the same procedure.

This is because the small number of cases in investigational treatments makes it impossible to generalize with respect to the impact these unexpected complications have on patients' quality of life when they occur. Whether existing cases are likely to be common or representative may not yet be clear.

Given the above, the clinician is unable to say yet whether, on balance, taking the risk is likely to be in a particular patient's best interests. So, the patient cannot “outsource” this assessment to assume the typical experience is likely to be her experience. The typical experience is not known.

Consequently, an additional, discrete section must be included as part of the information form, which will form the basis of directed “vivid consideration” in the decision‐making process. In this part of the discussion, the patient must be prompted to think vividly about what the unexpected side effect would mean for her life, were it to occur. Ideally, the patient would be given the opportunity to talk to someone who had experienced the unexpected side effect, or an expert in patient experiences of the side effect. Including a surrogate as part of this discussion should be encouraged.

The augmented consent process for novel DBS treatments would therefore include an additional section, which would be updated whenever a new significant unexpected complication occurs (Box 2).

**Box 2.**

**Serious adverse complications associated with investigational DBS of [target area] for [neurological or psychiatric condition]**

- [Side effect]: [current incidence]

The small number of patients who have undergone DBS of [target area] for [neurological or psychiatric condition] make it difficult for predictions to be made about precisely how likely it is that you will experience this complication. It is also difficult to determine the typical experience of [side effect] and its typical impact on the quality of life of patients who undergo DBS of [target area] for [neurological or psychiatric condition]. We, therefore, urge you to discuss with your physician and a friend or relation how [side effect] would impact your life in particular. If you would like, and if possible, you may request a meeting with an expert in patient experiences of [side effect].

When the likelihood that a significant adverse complication (such as seizures) will occur exceeds a certain threshold (e.g., 20% of patients), the vivid consideration exercise should remain part of the consent process. This should be so even when the number of cases is sufficient to determine the typical impact it has on the quality of life of patients.

## Augmented Consent in Practice

Having highlighted the ethical challenges of novel DBS procedures consideration needs to be given to how these challenges might be addressed in practice and to outline a practical approach to seeking “augmented” consent. We outline the procedures we recommend for adoption in the following case example. This case example involved treatment with a novel device for a novel indication. What follows demonstrates the application of the more general principles we outlined above. Full details of the augmented consent process in this particular case are provided in Appendix.

Patient 4 was considered for implantation of a programmable impulse generator that would allow seizure threshold to be monitored at the same time as delivering stimulation. The clinical rationale was that this device, the Activa PC&S (Medtronic), normally reserved for investigational use in clinical trials, would allow direct and more accurate measurements of seizure threshold. This in turn would allow for accurate safe dosimetry of standard stimulation parameters. The implant was approved for clinical use on the basis of compassionate use in an exceptional circumstance by the MHRA, UK.

This was a novel procedure. It carried the known risks associated with standard DBS procedures together with a number of unknown risks that by their nature were unpredictable. The thrust of the augmented consent process was to ensure that the patient was aware of these unknown risks and understood the possible implications for her. Although this process did not represent an assessment of mental capacity, the test of capacity set out in the Mental Capacity Act (2005) provided a helpful framework.

In the first phase of the process, the patient was given a bespoke information sheet and asked to study it in detail and to discuss it with their significant other. The information was presented in plain English and included:

- a brief description of the procedure
- the risks of proceeding
- the risks of not proceeding
- the benefits of proceeding
- the benefits of not proceeding

A copy of the information sheet is provided in Appendix. The document drew attention to the risks associated with standard DBS procedures but drew attention to the unknown risks that were attached to the novel procedure.

In order to ensure that the patient had fully understood and weighed the information concerning the procedure, three consultations were planned in order to check her retention of the information, her understanding and the consistency of her responses. These consultations included time with the patient alone and also with their significant other to establish their view on the decision. In order to minimize the burden associated with traveling to the neuroscience center, two of these consultations were conducted face to face via an Internet video call.

The consultation comprised a brief interview with the patient, which included direct questioning about:

- her understanding of the procedure
- her recall of the risks and benefits of proceeding or not proceeding
- her recall of the issue of unknown risks
- her decision about whether or not she wished to proceed.

Each session was documented in detail and the outcome shared with the neurosurgical team. As outlined above, particular attention was paid to her vivid understanding of the procedure and unknown risks. In this case, she demonstrated reliable recall of the detail of the procedure and the risks associated with it.

## Conclusion

Innovative neurosurgical treatments present a number of known risks, the natures and probabilities of which can be adequately communicated to patients via the standard procedures governing obtaining informed consent. However, they also come with unknown risks, which require an augmented approach to the consent process. The case study of ACC DBS for the treatment of chronic pain illuminates the complicated ethical issues in this regard. The uncertainty over the nature of the risks and their probability makes the determination of best interests more difficult, and underscores the need for the patient to make a rational, autonomous decision with respect to her treatment. The augmented consent process we outline would improve the quality of patient consent in the present case, and also in other innovative applications of DBS where risks are uncertain.

## Authorship Statements

Drs. Maslen, Savulescu, Cheeran, Aziz, and Mr. Pycroft were involved in the discussion that triggered the paper and the conceptual design of the paper. Drs. Maslen and Cheeran drafted the paper, with input from Dr. Prangnell. Drs. Aziz, Cheeran, Prangnell Green, Fitzgerald, and Boccard were involved in clinical care and outcome data collection. Drs. Pugh, Savulescu, and Aziz collaboratively reviewed and edited the manuscript for intellectual content. Drs. Pugh and Savulescu made substantial contributions to the ethical analysis in the paper. All co‐authors have all approved the final version.

## COMMENTS

This paper presents an important warning to our field to beware of the adverse effects of new neuromodulatory procedures. While the common view of DBS is that it is safe and reversible, brain circuits may undergo plastic changes that may not be reversible. It is just too early to know. Therefore this careful approach to obtaining informed consent for new indications and new brain targets is absolutely necessary.

Zelma Kiss, PhD

*Calgary, Alberta, Canada*

***

The authors highlight some of the ethical tensions that can ensue when offering novel treatments with uncertain benefits and side‐effects to patients with severe neurological disorders. They highlight the importance of a more detailed consent process that includes specific discussion regarding the potential ramifications of potential risks to patients' values and future goals.

Cynthia Kubu, PhD

*Cleveland, OH, USA*

Comments not included in the Early View version of this paper.

## Appendix

**Table:**

**Information Sheet** This information sheet provides details for the procedure that you are considering, together with the risks and possible benefits of this procedure. **Procedure** The procedure that has been proposed is: *Insertion of Medtronic PC&S Primary Cell Implanted Pulse Generator* The device will provide deep brain stimulation and also enable measurement your seizure threshold (i.e., the point at which increasing stimulation risks causing a seizure). The device will be used to measure the level of stimulation that you are likely to be able to tolerate without a seizure. The procedure to remove the old battery and replace it with the new one will involve being given an anaesthetic. This may be a general anaesthetic, or alternatively some local anaesthetic will be given along with drugs to make you sleepy. The anaesthetist will discuss which is more suitable for you with you before surgery. Once this device is removed the new device will be inserted and connected to the leads that you already have in place. After the operation, you will spend up to five days in hospital. While you are in hospital the DBS team will work with you to identify the best settings for your new device. This will involve up to two programming/threshold finding sessions per day with a specialist nurse or Dr. Cheeran. **Risks of Proceeding** ACC DBS is a novel procedure. This IPG has not been used to measure seizure thresholds in humans before, although more than 50 have been implanted in patients with Parkinson's Disease for other reasons. There are a number of known risks, which are set out below. It is also possible that the procedure might result in complications that cannot be predicted (so called “unknown unknowns”). This means it is not possible to list all the possible risks. Only the known risks are listed here. Seizure following anaesthesia Recurrence of seizures despite stimulation below detected seizure threshold Device fails to assess seizure threshold Pneumonia: 0.4–0.6% Infection: 2.8–6.1% *(if the IPG becomes infected the infection will be treated but the IPG will be removed and it will not be possible to replace it with another device)* Skin erosion: 1.3–2% Death **Risks of Not Proceeding** Current IPG device remains switched off No possibility of pain relief from IPG No change in frequency of seizures No reduction in pain relieving medication **Benefits of Proceeding** Possibility of pain relief with associated improvement in quality of life and day to day functioning Improved monitoring of seizure threshold which may mean that seizures can be reduced Possibility of reducing dosage of pain relieving medication **Benefits of Not Proceeding** Avoid known risks of surgery Avoid “unknown” risks
